# Improved Discrimination of Visual Stimuli Following Repetitive Transcranial Magnetic Stimulation

**DOI:** 10.1371/journal.pone.0010354

**Published:** 2010-04-28

**Authors:** Michael L. Waterston, Christopher C. Pack

**Affiliations:** Montreal Neurological Institute, Montreal, Quebec, Canada; Rutgers University, United States of America

## Abstract

**Background:**

Repetitive transcranial magnetic stimulation (rTMS) at certain frequencies increases thresholds for motor-evoked potentials and phosphenes following stimulation of cortex. Consequently rTMS is often assumed to introduce a “virtual lesion” in stimulated brain regions, with correspondingly diminished behavioral performance.

**Methodology/Principal Findings:**

Here we investigated the effects of rTMS to visual cortex on subjects' ability to perform visual psychophysical tasks. Contrary to expectations of a visual deficit, we find that rTMS often improves the discrimination of visual features. For coarse orientation tasks, discrimination of a static stimulus improved consistently following theta-burst stimulation of the occipital lobe. Using a reaction-time task, we found that these improvements occurred throughout the visual field and lasted beyond one hour post-rTMS. Low-frequency (1 Hz) stimulation yielded similar improvements. In contrast, we did not find consistent effects of rTMS on performance in a fine orientation discrimination task.

**Conclusions/Significance:**

Overall our results suggest that rTMS generally improves or has no effect on visual acuity, with the nature of the effect depending on the type of stimulation and the task. We interpret our results in the context of an ideal-observer model of visual perception.

## Introduction

Transcranial magnetic stimulation (TMS) is a powerful, non-invasive method of reversibly altering cortical function. The technique works by inducing a weak electrical current in a brain region that can be selected based on the placement of a magnetic coil near the scalp of the subject. Because it is safe and relatively painless the method has found increasing utility as a clinical tool for treating conditions such as Parkinson's Disease and depression [1], as well as in aiding rehabilitation following stroke [2]. Moreover, TMS is used widely in basic science investigations as a means of inferring the roles of specific brain regions in perception and behavior [3].

Repetitive TMS (rTMS) involves the application of a series of magnetic pulses over a period of seconds or minutes, with direct effects that last up to an hour [4] and clinical improvements that can accumulate over weeks [5]. These effects have been observed primarily in humans through indirect measures of cortical excitability, such as the threshold and amplitude of motor evoked potentials following stimulation of motor cortex [6] and the phosphene threshold following stimulation of visual cortex [7]–[8]. Such studies typically find reduced cortical excitability following low-frequency (1 Hz) stimulation and increased excitability following high-frequency (≥10 Hz) stimulation. More recently, two variations of a high-frequency stimulation protocol known as theta-burst have been shown to cause reduced (continuous theta-burst) or increased (intermittent theta-burst) excitability [4].

A more direct measure of the effects of rTMS comes from neurophysiological studies conducted in anaesthetized cats. Consistent with the notion that low-frequency stimulation reduces cortical excitability, Allen et al. [9] found decreased spike rates for over 5 minutes following two-second trains of rTMS at 1, 4, and 8 Hz stimulation. Similarly, EEG recordings from the anaesthetized cat also show decreased visually evoked potentials following 1 Hz and 3 Hz rTMS and increased potentials following 10 Hz stimulation [10].

Given the consistency of the effects of rTMS across brain areas and measures of excitability, one might expect to find predictable effects of rTMS on performance during psychophysical or behavioral tasks. Indeed the observation that certain rTMS protocols lead to reduced cortical excitability has led to the notion that these protocols create “virtual lesions” in the targeted brain region [11], [12]. Thus it is surprising that functional measures following rTMS often yield a rather inconsistent pattern of results [13], [14], [15], [16].

Although the apparent discrepancy between rTMS-induced effects on cortical excitability and those on behavioral performance may appear puzzling, it is important to recall that gross measures of neuronal population activity need not correlate with performance on a given task. In the visual system in particular there are many examples in which stimuli that can be expected to increase visual responses decrease perceptual performance (e.g., Tadin et al. 2003 [17]). Indeed an important function of visual cortical networks is to generate responses that represent important or unusual features of the visual input [18]. Such “sparse coding” leads naturally to a reduction in overall cortical activity, but improved discrimination of the features encoded by the population [19].

In this work we have investigated the effects of various rTMS protocols on performance on tasks that require the observer to discriminate the orientation of a visual pattern. We chose this task because it is known that many cells in primary visual cortex can be preferentially excited by visual stimuli of specific orientations [24]. By matching the task closely with the tuning of the underlying neurons we hoped to more directly measure the functional effect of rTMS. In contrast to expectations of a “virtual lesion” we find that rTMS of the visual cortex often leads to improved visual discrimination performance, and that these improvements last for many minutes following stimulation. We interpret our results in the context of statistical models in which the overall level of excitability is less important than the pattern of activity across the neuronal population for predicting psychophysical performance [20]. If our interpretation is correct, it may be useful for understanding the role of therapeutic stimulation, especially for disorders commonly attributed to visual cortex such as amblyopia [21] or migraine [22].

## Materials and Methods

### Subjects

During the three experiments reported here, a total of 27 subjects were tested (mean age 24 years, 15 male, 12 female). All subjects were naïve as to the aims of the experiment and were recruited via online advertisements. All subjects had normal or corrected-to-normal vision and were compensated at the rate of $50 per session. Subjects were excluded from the study if they had metal implants, prostheses, family history of seizure, were pregnant, or were prescribed antidepressant medications. Most subjects participated in three sessions, each of which lasted approximately two hours. All aspects of the recruitment procedures and experimental protocols were approved by the Ethics Review Board of the Montreal Neurological Institute. Written informed consent was obtained from all subjects.

### Visual Cortex Localization

#### V1 localization

Primary visual cortex (V1) was located by testing for phosphenes in a 3 by 3 grid pattern, spaced 1 cm apart, centered 2 cm to the left and 4 cm above the inion. If phosphenes could not be evoked at any of those locations the center location was used. Subjects fixated at a central red point on a black screen with a faint 1 cm grid so that they could indicate the phosphene location. A pair of pulses 50 ms apart at 80 percent of maximum stimulator output was used to evoke the phosphenes [23].

### rTMS Stimulation

For all experiments, TMS was administered with a Magstim Rapid 2 stimulator with dual power supply units. The air-cooled figure-eight 70 mm coil was designed to deliver maximum stimulation at the overlap of the two sides of the coil. Offline stimulation was used for all experiments.

Continuous theta-burst TMS was delivered as five bursts of three 50-Hz pulses every second for 40 seconds, for a total of 600 pulses. Theta-burst stimulation was delivered at 43 percent of the maximum single-pulse stimulator intensity (the highest intensity at which the stimulator could reliably produce the theta-burst sequence). Low-frequency stimulation was delivered as one pulse every second for 20 minutes, for a total of 1200 pulses. Low-frequency stimulation was delivered at the subject's phosphene threshold at a mean of 64.6% of maximum stimulator output. Phosphene thresholds were determined by stimulating over V1 as localized in the procedure described above (see *V1 localization*). The stimulator output was decreased in steps of 2 percent stimulator output from 80 percent of maximal output until the subject reported phosphenes for 2 out of 4 stimulations.

### Visual stimuli and procedure

#### Psychophysics environment

Stimuli were generated with the Psychophysics Toolbox version 3.0.8 extension for Matlab (version 7.4.0) running on an Apple Mac Pro with an nVidia GeForce 7300 GT video card. The display was a Trinitron A7217A CRT monitor with a refresh rate of 75 Hz. Gabor patterns were presented with the same space-averaged luminance as the background (4.75 cd/m2 as measured by a Konica Minolta LS-110 luminance meter). The color lookup table of the video card was restricted through Psychophysics Toolbox to the luminance range of the stimulus. This increased the number of distinct grey levels that could be displayed. The minimum contrast step was measured by the luminance meter to be 0.5% Michelson contrast. Fixation was monitored with an SR research Eyelink 1000. If the gaze deviated more than 2 degrees from the fixation point during any of the experiments a tone was sounded and the trial was repeated. Subjects viewed the stimuli with their heads fixed in a chin rest with forehead support 57 cm from the display and indicated their responses with a Microsoft Sidewinder game pad. Responses were recorded with the Psychophysics Toolbox game pad module.

#### Experiment 1: Coarse orientation discrimination, single location

**Figure 1:**
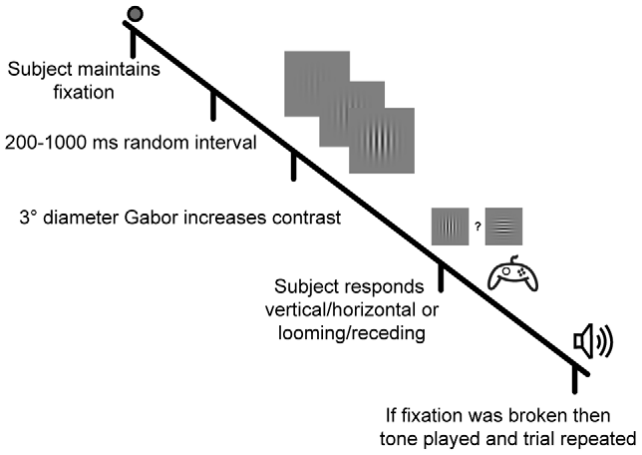
Experimental procedure. On each trial subjects maintained fixation and indicated with a button press the perceived orientation of a Gabor pattern that was presented for 27 ms (static stimulus condition) or gradually increasing in contrast (reaction time experiment). For coarse orientation discrimination, subjects indicated whether the Gabor patterns appeared to be vertical or horizontal. For fine orientation discrimination, subjects indicated whether the Gabor pattern was oriented to the left or right of vertical.

In baseline testing a low-contrast Gabor grating (3 degrees in diameter) with spatial frequency of 0.75 cycles per degree was flashed for 27 ms (2 frames) at 6 degrees to the right of fixation (Figure 1). Subjects performed a two-alternative forced choice task, indicating after each stimulus presentation whether the Gabor patch was oriented vertically or horizontally. Contrast was adjusted via a staircase procedure until a criterion level of 75% accuracy was reached. The Michelson contrast started at 11% and was adjusted down by 0.5% after every 3 correct responses and up by 0.5% after every incorrect response. The threshold was calculated as the mean of the last 20 reversals. The contrast was then fixed, and the same stimulus was tested 400 times both before and after theta-burst rTMS. Eight subjects were tested with theta-burst stimulation over primary visual cortex and over the vertex as a control in a darkened room with eyes closed. We excluded the results from one subject who was unable to maintain fixation.

#### Experiment 2: Coarse orientation discrimination, multiple locations

To investigate the distribution of the change in acuity across the visual field over time Gabor gratings were presented with an ascending method of limits procedure in which contrast increased smoothly from zero at an exponential rate. Subjects were instructed to respond as soon as they were able to accurately perceive the orientation of the stimulus. The stimulus was presented sequentially and predictably in nine different locations in the visual field (three ipsilateral to stimulation and six contralateral to stimulation). Five subjects were tested with 1 Hz stimulation over primary visual cortex and the vertex, and a different set of eight subjects was tested with theta-burst stimulation over primary visual cortex and the vertex. We again excluded the results from one subject who was unable to maintain fixation for the duration of the experiment. For comparison, Table 1 describes the main conditions for all three experiments.

**Table 1:** Characteristics of experiments.

	Experiment 1	Experiment 2	Experiment 3
**Orientation Discrimination**	Coarse (90 degrees)	Coarse (90 degrees)	Fine (<5 degrees)
**Stimulus Locations**	6 degrees to right	6 contralateral, 3 ipsilateral	6 degrees to right
**Stimulus Presentation**	Static	Increasing Contrast	Static
**Subjects**	7	7 theta-burst, 5 1-Hz	6
**TMS Sequence**	40 seconds theta-burst	40 seconds theta-burst, 20 minutes 1-Hz	40 seconds theta-burst
**Average TMS Energy**	600 pulses at 43%	600 theta-burst pulses at 43%, 1200 1-Hz pulses at average 65%	600 pulses at 43%
**TMS Control Site**	Vertex	Vertex	None

#### Experiment 3: Fine orientation discrimination, single location

Fine orientation discrimination was tested by presenting the Gabor gratings 6 degrees to the right of fixation for 27 ms oriented either slightly to the left or slightly to the right of vertical. Subjects were given the forced-choice task of indicating whether the grating was tilted to the right or to the left. The size of the tilt was adjusted with a staircase procedure so that individual performance in baseline testing approached 75% correct. The same tilt magnitude was then tested 400 times before and after theta-burst rTMS stimulation. Six subjects were tested with 40-second theta-burst stimulation over primary visual cortex in a darkened room with eyes closed. Vertex stimulation was not tested in this condition.

#### Data Analysis

For the experiments with constant stimuli, the average percentage correct in 400 pre-stimulation trials was compared to the average percentage correct for the first 400 post-stimulation trials in the same subjects. Statistical significance was tested with a chi-square test for homogeneity of proportions and maximum p-value of 0.05. Changes in group before versus after stimulation means were tested with repeated measures t-test of the two sets of subject means and maximum p-value of 0.05. For experiments with the ascending method of limits reaction time procedure, z-scores were calculated for each testing location for each subject. Post-stimulation reaction times were calculated as deviations from pre-stimulation mean reaction time divided by pre-stimulation standard deviation performance for each location. All calculations were performed with the SciPy library for Python.

## Results

Our goal in these experiments was to characterize the effects of repetitive transcranial magnetic stimulation (rTMS) on performance on various tests of visual perceptual acuity. In particular we were interested in determining how rTMS delivered at different frequencies changed performance on several discrimination tasks and how these changes were distributed in space and time.

### Experiment 1: Coarse orientation discrimination, single location

Most neurons in the primary visual cortex (V1) are selective for the orientation of a visual stimulus, and these neurons project to extrastriate cortical areas that are known to be involved in forming perceptual decisions [24]. Thus to the extent that rTMS influences neuronal activity, we might expect the accuracy or speed of decisions about stimulus orientation to be affected by stimulation of V1.

In the first experiment, we examined the effects of rTMS on subjects' ability to report the orientation of a briefly presented stimulus. The stimulus was a small Gabor patch presented 6° to one side of the fixation point. The orientation of the Gabor patch could be horizontal or vertical, and it was displayed for 27 ms. Subjects were then required to report the orientation of the stimulus with a button press.

In preliminary testing we determined the contrast of the Gabor stimulus that yielded roughly 75% correct performance for each subject. We then tested the percent of correctly identified orientations during 400 presentations of the threshold-contrast stimulus. The 400 presentations were repeated after applying 40 seconds of theta-burst stimulation (see Methods for stimulation protocol) to either the primary visual cortex or to the vertex of the scalp. This stimulation protocol is thought to exert inhibitory effects on the targeted brain region [4].

**Figure 2:**
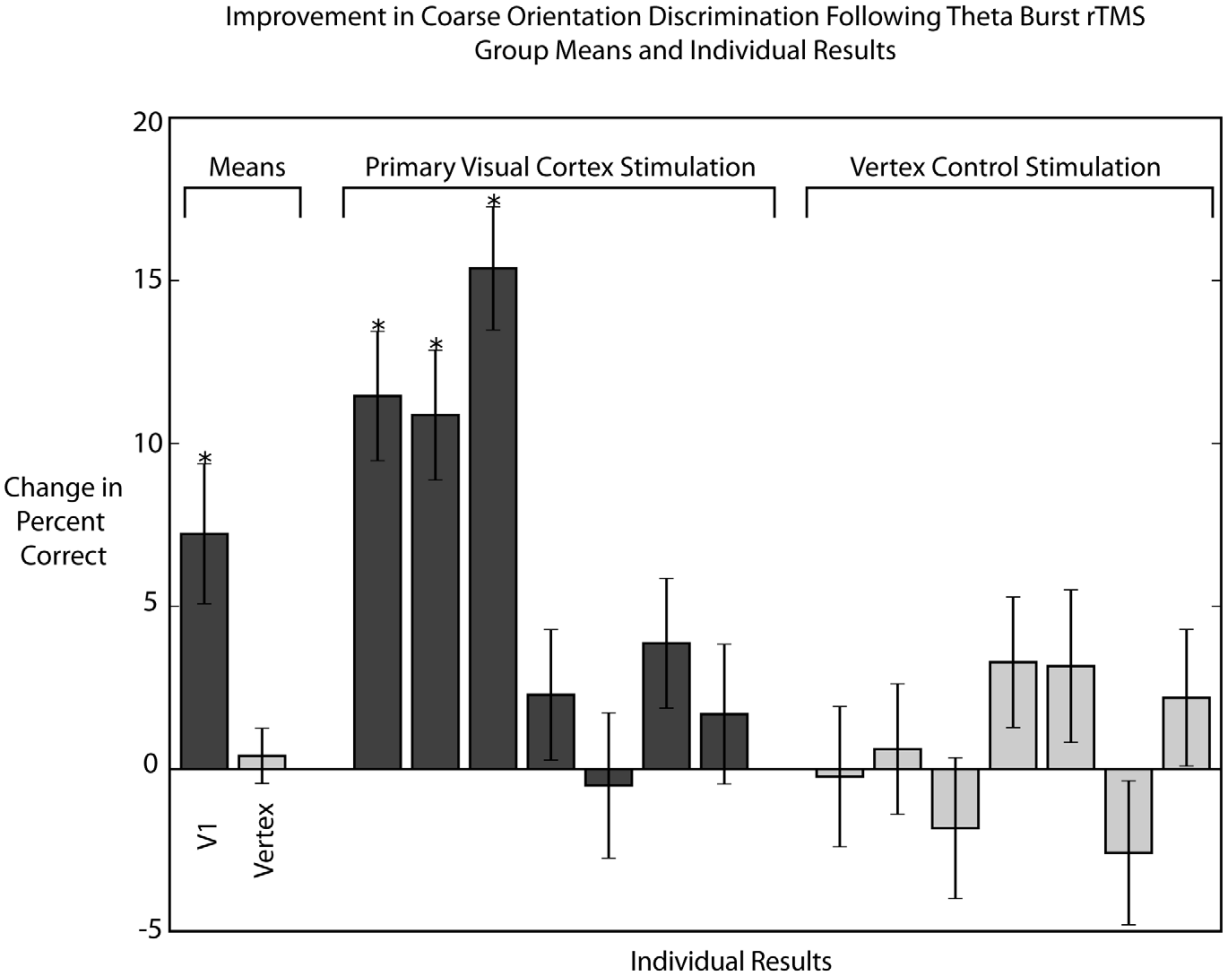
Effects of theta-burst stimulation on coarse orientation discrimination. Following stimulation of the primary visual cortex (dark gray bars), the percentage of correct orientation judgments increased by an average of 7.2 percent (left-most bar). Stimulation of the control site at the vertex of the scalp (light gray bars) did not on average improve performance.

Figure 2 shows the results for 7 subjects. Despite the inhibitory nature of the rTMS protocol, post-rTMS performance in discriminating orientation improved in 6 out of 7 subjects (dark gray bars, Figure 2), and in 3 subjects this improvement was statistically significant (chi-square test for homogeneity of proportions, p<0.05). The improvement was also significant (p = 0.034) for the group mean (left-most bar, Figure 2), with performance increasing from an average of 72.4% pre-TMS to 78.9% post-TMS. No obvious difference was found between responders and non-responders in terms of phosphene threshold, dominant eye, sex, or age. However, the change in performance showed a strong negative correlation with pre-TMS performance (linear regression, p<0.02), suggesting that subjects who found the task more difficult exhibited greater improvements. Overall contrast sensitivity (as measured by the pre-TMS contrast threshold) did not correlate well with changes in performance (linear regression, p>0.3).

These results could be related to an effect of rTMS on neuronal responses in the visual cortex, as the theta-burst protocol in particular has been shown to affect neuronal excitability and short-term plasticity in a variety of other contexts [4]. However, an alternative explanation is that rTMS influenced psychophysical performance indirectly by increasing alertness, arousal, or some other physiological response that was not specific to visual cortical stimulation. To control for this possibility, we also tested the same subjects but with rTMS targeted to brain regions beneath the vertex of the scalp, rather than the visual cortex (in counterbalanced order). In this case no subject showed a statistically significant (chi-square test for homogeneity of proportions, p<0.05) change in performance (dark gray bars, Figure 2). For the group mean performance was 74.9% before rTMS and 76% afterwards, which was not significant (p>0.14), and there was no significant correlation between the change in performance and pre-TMS accuracy (p>0.16). Thus we conclude that the effects of rTMS on visual discrimination performance for static stimuli are not a direct consequence of the stimulation protocol *per se*, but rather are specific to the targeted brain region.

### Experiment 2: Coarse orientation discrimination, multiple locations

The previous section demonstrated that performance on a coarse orientation discrimination task improved following the application of 40 seconds of theta-burst rTMS. These results (Figure 2) represent the average performance over a 10-minute, post-rTMS period during which the test stimulus was shown repeatedly at a single retinal location. In the next set of experiments we sought to determine, for those subjects who showed improved performance, how the improvements varied over space and time. This required designing a stimulus that could rapidly probe visual acuity across multiple retinal locations. This allowed us to map the extent of the rTMS effects, although the stimulus differences precluded a direct comparison with the results from the first experiment.

**Figure 3:**
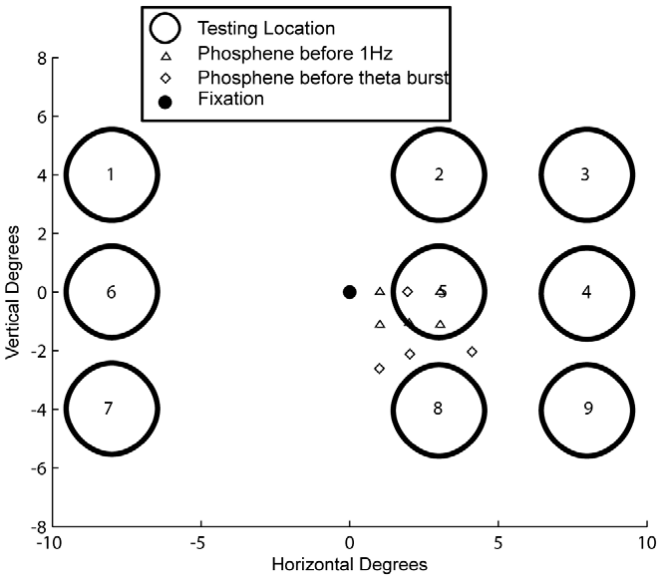
Stimulus and phosphene locations for the reaction-time task. Coarse orientation discrimination was tested at nine locations in the visual field (open circles). Subjects fixated at the central point (black dot) as orientation discrimination was tested in each of the locations in order. Before stimulation, phosphenes were elicited in the right visual field. For the subjects that were able to report the specific location of the phosphene, they are marked by the small triangles and diamonds. All phosphenes were contralateral to stimulation and clustered around the horizontal meridian (location 5).

Figure 3 shows the layout of the experiment. Orientation discrimination was tested at nine locations (large circles) in the visual field, and the small colored dots in the figure show the locations of the evoked phosphenes, which were always contralateral to the site of stimulation. In order to map the strength of the improvement rapidly, we used a reaction-time task in which the stimulus appeared at one of the nine locations and gradually increased in contrast (see Methods and Figure 1). The subject's task was to press a button as soon as he or she could accurately determine the stimulus orientation, and this reaction time was used as a measure of contrast sensitivity. From trial to trial the stimulus location changed predictably in the pattern indicated by the numbered circles in figure 3. This paradigm proved to be an efficient method of characterizing the effects of rTMS across the visual field, as we were typically able to test all nine locations in less than one minute.

As in the previous experiment, we determined a baseline level of performance by testing subjects on the reaction-time discrimination task prior to the application of rTMS. For each subject we obtained a distribution of reaction times and quantified the post-rTMS performance over one hour in terms of a z-score relative to the baseline distribution of each individual testing location. In addition to the theta-burst stimulation protocol used in the previous experiment, we also tested subjects following low-frequency (1 Hz) stimulation, which is also thought to inhibit neuronal activity in the targeted brain region [9]. Mean accuracy across subjects was above 95% both before and after rTMS.

**Figure 4:**
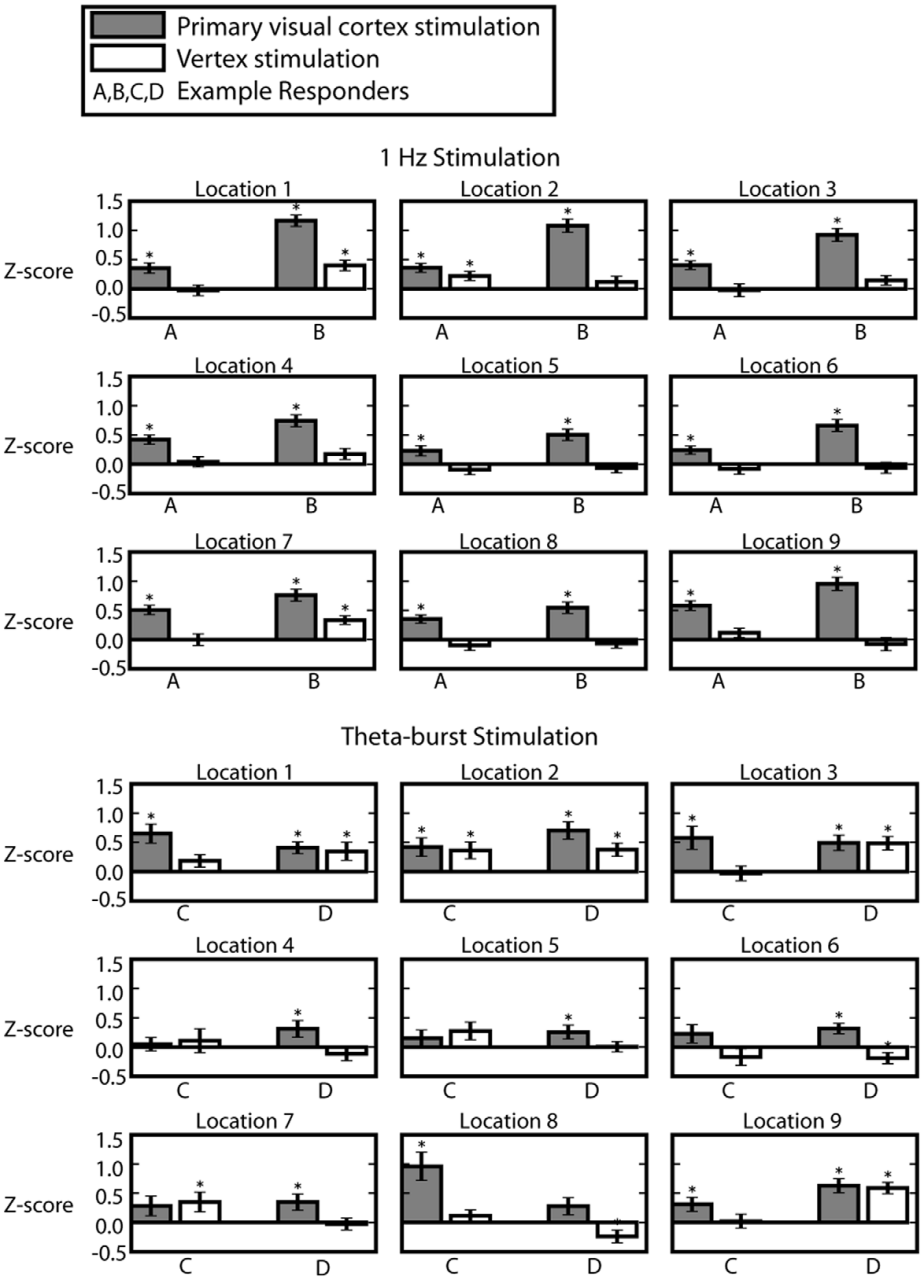
Effects of rTMS across visual space for two subjects in each of the two stimulation (1 Hz and theta-burst) conditions applied to the occipital cortex (gray bars) or the vertex of the scalp (white bars). Subjects were required to indicate whether a Gabor pattern was oriented horizontally or vertically, and the response time was taken as a measure of performance. Each panel shows the change in response time, which is represented as a z-score relative to the distribution of response times seen in pre-rTMS testing. Here positive numbers represent faster reaction times.

Following 1 Hz stimulation of visual cortex, 4 out of 5 observers showed a statistically significant (p<0.05, independent t-test) improvement relative to the vertex control. For theta-burst stimulation, 4 out of 7 observers showed significant improvements. An additional two subjects showed significantly more improvement in the vertex condition than in the occipital stimulation condition, possibly suggesting an effect of theta-burst stimulation on motor responses [14]. In order to isolate the effects of occipital rTMS across visual space, we calculated the changes in performance at each of the 9 tested locations. Figure 4 illustrates the extent of the improvement in visual space for four subjects (all showing strong effects of occipital stimulation) following theta-burst and 1 Hz stimulation. Each panel presents the change in performance at a particular testing location. Locations 1, 4, and 7 were ipsilateral to stimulation and 2, 3, 5, 6, 8, and 9 were contralateral to stimulation. Surprisingly, improvements on the orientation discrimination task following stimulation of the primary visual cortex (gray bars) are found throughout visual space, with no obvious bias toward the contralateral or ipsilateral visual field.

**Figure 5:**
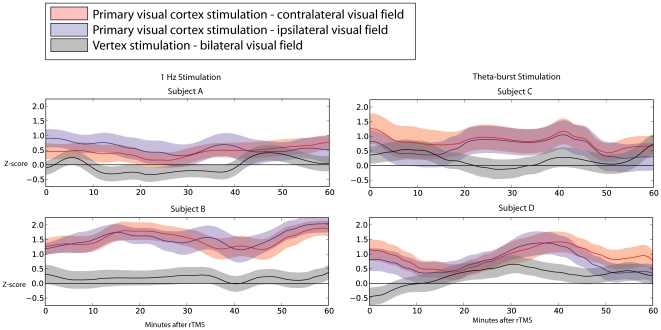
Time-course of results in the reaction-time task. Each panel shows the time course of results for the representative subjects in figure 4 following 1 Hz (left) or theta-burst (right) stimulation. For all subjects a modest but consistent improvement in both ipsilateral (blue) and contralateral (red) visual fields is closely related throughout the time course for both theta-burst and 1 Hz stimulation as compared to stimulation of the vertex of the scalp (black). The improvement persists beyond 60 minutes post stimulation.

Figure 5 shows the time-course of the change in visual discrimination performance following rTMS for the same subjects shown in Figure 4. Here performance is quantified as the mean z-score for 1 Hz (left) and the theta-burst (right) stimulation protocols in smoothed 1-minute bins post-TMS for a period of one hour. Each panel shows the effects on contralateral (red lines) and ipsilateral (blue lines) stimulus locations following visual cortex stimulation, as well as the effects of vertex stimulation (black lines) for both visual hemifields combined. Although there is some variability (due partially to the smaller subject pool), the correlation between ipsilateral and contralateral performance was consistent over time, and the improvement in these responders persisted beyond 60 minutes after stimulation.

### Experiment 3: Fine orientation discrimination, single location

Our results up to this point suggest that rTMS can improve performance on tasks that require coarse orientation discrimination (horizontal versus vertical). This conclusion holds for different measures of discrimination performance and stimulation protocols. The next experiment was designed to assess whether the effects of rTMS generalized to fine discrimination tasks, which are likely to rely on different features of the neuronal population response [25].

The design of this experiment was similar to that of the first experiment. Subjects were asked to indicate with a button press their perception of the orientation of a briefly presented Gabor patch. However, in contrast to experiment 1, the goal here was to indicate whether the stimulus was tilted to the left or right of vertical. The Gabor patch was presented at a constant contrast, which was low but consistently perceptible (Michelson contrast of 25%). The direction of tilt was varied randomly from trial to trial, and the magnitude of the tilt was determined for each subject as the value that led to 75% correct performance in preliminary testing.

**Figure 6:**
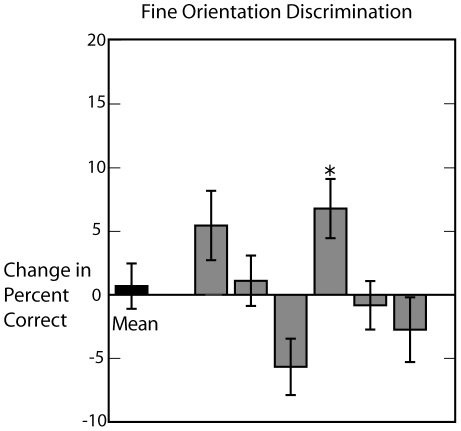
Results for the fine orientation discrimination task. Subjects were required to indicate whether the orientation of a briefly-presented Gabor stimulus was to the left or right of vertical. The bars show the effects of continuous theta-burst stimulation of primary visual cortex on six subjects and for the mean (leftmost bar).

Figure 6 shows the resulting change in performance following theta-burst rTMS in 6 subjects. While some subjects improved their fine orientation discrimination following theta-burst stimulation, the improvement in responders was smaller than that observed with coarse orientation discrimination (Figure 2), and the overall effect for the group was not significant (dependent t-test p = 0.74). Of six subjects tested, one had a significant improvement in the percentage of correctly identified trials (chi-square test for homogeneity of proportions, p<0.05). As in the coarse discrimination task, the effects of rTMS were more strongly correlated with the task difficulty (linear regression, p = 0.11) than with the pre-stimulation discrimination threshold (linear regression, p = 0.31), though in this case neither effect was statistically significant. Overall the difference in performance on the two tasks was marginally significant (p = 0.09). Of course it is possible that a smaller effect on the fine discrimination task might have been detected with a larger subject pool, but a comparison of Figures 2 and 6 suggest that the effects of rTMS are stronger for coarse than for fine discrimination tasks.

## Discussion

We have tested human subjects on several tasks requiring psychophysical discrimination of visual stimulus features following the application of repetitive transcranial magnetic stimulation (rTMS). In most cases in which rTMS was applied to the visual cortex, coarse discrimination performance improved for both theta-burst and low-frequency (1 Hz) stimulation. In both cases the improvements were found across the visual field and lasted for many minutes following rTMS. Taken together, these results suggest that rTMS can improve performance on visual psychophysical tasks, with both the magnitude and the nature of the improvements being dependent on the task and the stimulation protocol.

### Comparison to previous work

**Figure 7:**
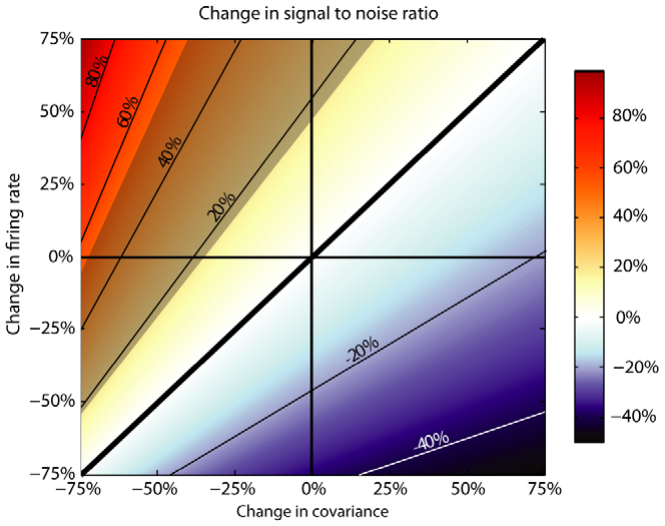
Theoretical analysis of possible rTMS-induced changes in neuronal responses. Each pixel in the map represents a change in signal-to-noise ratio (see text for mathematical definition) for a given change in firing rate (ordinate) or covariance in neuronal responses (abscissa). Larger signal-to-noise ratios are indicated by reddish colors, and smaller ratios are indicated by bluish colors. Although decreasing firing rates leads to poorer signal-to-noise ratio, such decreases can be compensated for by a corresponding decrease in covariance. The observed mean improvement in orientation discrimination following theta-burst stimulation (72.4% pre-TMS to 78.9%+/−3.6% post-TMS) corresponds to an 18%–53% improvement in signal-to-noise ratio (highlighted gray area).

Relative to the large body of work on the effects of rTMS on motor behavior, studies of rTMS in sensory perception are somewhat rare. rTMS of the primary visual cortex has been reported to diminish [13], [26] or to improve [21] visual acuity, depending on the nature of the task, the behavioral readout, and the frequency and location of stimulation. Thompson et al. [27] demonstrated a double dissociation for coherent motion perception following rTMS stimulation of V1 and V5/MT, suggesting competing percepts from different visual areas. Cattaneo et al. [28] reported an improvement in visual short-term memory when a single pulse was administered at the end of the memory period; however they also found an increase in reaction times when it was applied at the onset of the memory period. Our results are similarly sensitive to the experimental details: Improvements appear to be more common for coarse than for fine discrimination tasks and dependent on the stimulation frequency and task difficulty. As we show below (Figure 7), interpreting these complex results will require further neurophysiological studies of rTMS, as even modest changes in physiological parameters, such as overall excitability and noise correlations, can be expected to lead to large positive or negative changes in psychophysical performance.

One parameter that we have not addressed in our work is the activation state of the cortex during the application of rTMS. Silvanto et al. [29] reported that visual activity during high-frequency rTMS of primary visual cortex affected subsequent ability to discriminate visual stimuli. The nature of the effect depended on the similarity of the test stimulus to that presented during rTMS, suggesting that the effects of rTMS depend specifically on the level of activation of a given subpopulation of neurons. Similar results were obtained when offline rTMS was used to manipulate neuronal excitability prior to the application of online rTMS [30]. During stimulation for our static stimuli, subjects were in a darkened room with their eyes closed, and during stimulation for our reaction time experiments subjects were performing the discrimination task. The results in our experiments were similar, but it would be interesting to determine if the enhanced performance we have observed could be targeted by more specific stimulus conditions during rTMS.

In the extrastriate cortex, online rTMS of V3A has been studied for its role in motion perception along with area MT/V5 [31], [32]. Cowey et al. [31] found that online rTMS of both V5 and V2/V3 impaired direction of coherent motion discrimination but not detection of coherent motion. McKeefry et al. [32] used 5 pulses of 25 Hz rTMS delivered to either V5 or V3A during a reference and test moving stimulus to demonstrate a reduced perceived speed during stimulation. The same stimulation protocols did not affect the processing of spatial frequency, suggesting that the results of rTMS in the extrastriate cortex are specific to the presumptive functional role of the targeted area.

### Possible neurophysiological mechanisms

Our results may seem surprising in light of previous work showing that low-frequency and continuous theta-burst protocols are found to reduce activity of the stimulated brain region [4], leading to “virtual lesions”. In particular these protocols lead to decreased excitability for subsequent applications of TMS [7], [34], smaller amplitudes of evoked potentials [10], and suppression of evoked spiking activity [9], [35]. The latter results are particularly relevant to the current work, as they show that rTMS reduces the firing rates of single neurons in the visual cortex of the anesthetized cat.

Intuitively one might think that weaker responses of cortical neurons might lead to poorer visual discrimination acuity, but this is not necessarily true. A great deal of previous experimental and theoretical work has shown that coarse discrimination performance is limited both by the firing rates of individual neurons and the noise correlations among neurons [36], [37], [38], [39]. Indeed when evaluating the signal-to-noise ratio for the sum of a population of neurons, it is the correlation in the noise between individual cells that is the critical factor limiting any benefits from increased population size [20], and recent evidence from voltage-sensitive dye imaging of macaque primary visual cortex suggests that this correlation-dependent baseline variance dominates the population noise independent of the presence of stimuli [40]. Thus if rTMS reduces the correlated noise between neurons one might expect to find improved discrimination even in the presence of significantly reduced firing rates. The results of Pasley et al. [35] suggest an overall decorrelation of neural activity (as measured by phase locking of spikes to local field potentials), suggest that such a hypothesis is not unreasonable. Hamidi et al. [41] also find that improvements in working memory performance following 10 Hz rTMS are associated with a decrease in alpha-band EEG, consistent with a decorrelation of neural activity. Improved performance following high-frequency rTMS may thus be attributed to decreased noise correlation rather than increased cortical excitability. The different effects of high- and low-frequency rTMS on cortical excitability may be independent of their effect on performance.

To explore this idea further we calculated the signal-to-noise ratio for a population of neurons that fire in response to a stimulus and that are affected by correlated noise. This value for the sum of a population of *n* neurons with mean firing rate *x* can be expressed as [20]:
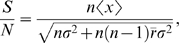
where *r* is the mean correlation among neurons and *σ^2^* is the variance of the population.

Figure 7 illustrates the effect of manipulating the excitability *X* and the correlation strength *r* on a population of 100 neurons with Poisson variance. The change in signal-to-noise ratio is independent of initial values for firing rate and variance. Because any increase in signal-to-noise ratio of additional neurons is negligible for population sizes greater than 100 and *r* greater than 0.05 [20], we take as our baseline the case when *r* = 0.12 and *n* = 100. Improved signal-to-noise corresponds to red colors, and decreased performance corresponds to blue colors. Importantly there is a portion of the parameter space (reddish colors in lower left quadrant) in which better signal-to-noise ratios are achieved despite reductions in overall excitability of the magnitude observed by Allen et al.[9].

In our experiments, following theta-burst stimulation of primary visual cortex (Figure 2), orientation discrimination increased from 72.4% to 78.9%+/−3.6% which corresponds to an increase in signal to noise ratio of 35%+/−18%. As shown in Figure 7 (gray area) a comparable improvement in signal-to-noise ratio can be obtained despite large decreases in firing rate, as the metric is quite sensitive to interneuronal correlation strength. This suggests that a plausible mechanism by which rTMS exerts its influence is via a long-lasting decorrelation in neuronal responses, in a manner similar to the effects of adaptation on V1 [42]. It also illustrates the point that a decrease in excitability need not translate into diminished function. In fact, decreasing firing rates may be intrinsically linked to decreased inter-neuronal correlations [43], which could explain why inhibitory manipulations improved performance in our experiments.

While the decorrelation hypothesis outlined above is a plausible explanation for the improved discrimination that we observed, other explanations are possible. With unilateral rTMS it can be argued that inhibitory stimulation disinhibits activity in the other hemisphere [44]. However, inter-hemispheric rivalry would be inconsistent with the bilateral visual field improvement that we observed in our results (Figure 4). Likewise, reducing activity in primary visual cortex could disinhibit other cortical areas that are better suited to the task being performed. However, these areas receive the bulk of their input directly or indirectly from V1 [45], so it is unlikely that the sensory information necessary to complete the task reaches higher visual areas without passing through the stimulated cortex. Alternatively, sensory discrimination can be thought of as a neural evidence accumulation problem where reaction time and accuracy are traded off [46]. In this model, inhibitory stimulation could delay reaction times to allow additional evidence to accumulate, or alternatively it could lower the accuracy threshold to allow for faster reaction times. However, the observed improvement in both reaction time (Figure 4) and accuracy (Figure 2) suggests that the quality of the sensory signal itself was enhanced. While recent neurophysiological data provides some basis for a hypothesis about the reason for the improvements ([9]; Figure 7), there is no obvious explanation for its spread in space and time. In our studies enhancements in visual performance lasted for approximately one hour and covered the entire bilateral area over which testing was carried out (Figure 4 and Figure 5). Finally, rTMS may be able to improve detection by interfering with cortical suppression. Discrimination of brief moving stimuli is paradoxically more difficult for larger rather than smaller stimuli [17]. This spatial suppression can be inhibited using offline 1 Hz rTMS of MT/V5, leading to improved motion discrimination of large (8 degree) stimuli [47]. It is possible that similar suppressive mechanisms were disrupted in our experiments; however such spatial suppression is strongest for large, high-contrast stimuli and we detected an improvement in the discrimination of moderately sized, low-contrast stimuli.

### Possible applications

Potential clinical applications of TMS have been investigated for many neurological conditions [1], [48]. For example, following 5 Hz rTMS stimulation of motor cortex, Parkinsonian patients demonstrated fewer velocity inversions in their pointing motion, indicative of a reduction in bradykinesia. Similar improvements have been found in patients suffering from depression [5], [49], [50], [51] and in those who are recovering from stroke [52], [53], [54]. To the extent that our speculation about the effects of rTMS on interneuronal correlations is correct, our results may also have implications for conditions in which neuronal synchronization is related to disorders of cognitive function [55].

Of particular relevance to the present work are some recent studies involving TMS of the visual cortex for the treatment of migraines [22], [56] and amblyopia [21]. The latter study in particular showed that, for subjects with amblyopia, 1 Hz and 10 Hz rTMS of primary visual cortex leads to temporary improvement in the acuity of the amblyopic eye [21]. A similar improvement was found in testing on subjects with normal vision. Our results suggest that protocols such as theta-burst may lead to even stronger improvements in the visual acuity of amblyopes, and future work will concentrate on testing this idea.
